# The effect of adsorbate induced surface strain on oxygen island formation on platinum surfaces

**DOI:** 10.1039/d5fd00161g

**Published:** 2026-04-29

**Authors:** Florian Nitz, Alexander Kandratsenka, Daniel J. Auerbach, Theofanis N. Kitsopoulos, Alec M. Wodtke

**Affiliations:** a Institute for Physical Chemistry, Georg-August University of Göttingen Germany; b Max Planck Institute for Multidisciplinary Sciences Germany alec.wodtke@mpinat.mpg.de; c School of Mathematics and Natural Sciences, University of Southern Mississippi Hattiesburg Mississippi 39406 USA; d International Center for Advanced Studies of Energy Conversion, Georg-August University of Göttingen Germany

## Abstract

The reactivity of heterogeneous catalysts under working conditions may be strongly influenced by adsorbate–adsorbate interactions which alter reaction barriers and lead to the formation of ordered adsorbate structures like islands. To predict catalytic reactivity, accurate knowledge of adsorbate–adsorbate interaction energies is required, but it is rarely available. One challenge arises from the surprisingly long range over which these interactions exert influence. We show in this work—using DFT with periodic boundary conditions and an (8 × 8) unit cell—that a single O-atom adsorbed at Pt(111) induces significant Pt atom displacements out to more than 7 Å. This adsorbate induced surface strain allows O*-atoms at distances of 14 Å to experience repulsion between one another due to the adsorbate induced displacement of the Pt atoms between them. Similar calculations using smaller unit cells overestimate repulsion due to interactions between O* atoms in neighboring periodic images. The use of an (8 × 8) unit cell removes this error, revealing short range attractive interactions between 3rd nearest neighbors. We have used these improved DFT interaction energies to perform kinetic Monte Carlo simulations of oxygen island formation and to show how the interplay of short- and long-range forces determines the sizes and shapes of these islands. Neglect of long-range interactions leads to round and compact island structures, which are in conflict with STM experiments. Including all interactions out to the 9th nearest-neighbor results in simulations that eerily resemble the STM observations.

## Abstract 2

Adsorption of a single oxygen atom on Pt(111) displaces the Pt surface atoms within 7 Å of the oxygen atom. These displacements result in long-range repulsion between adsorbed oxygen atoms and lead to a reorganization of the adsorbed layer at elevated coverages.

## Graphical abstract

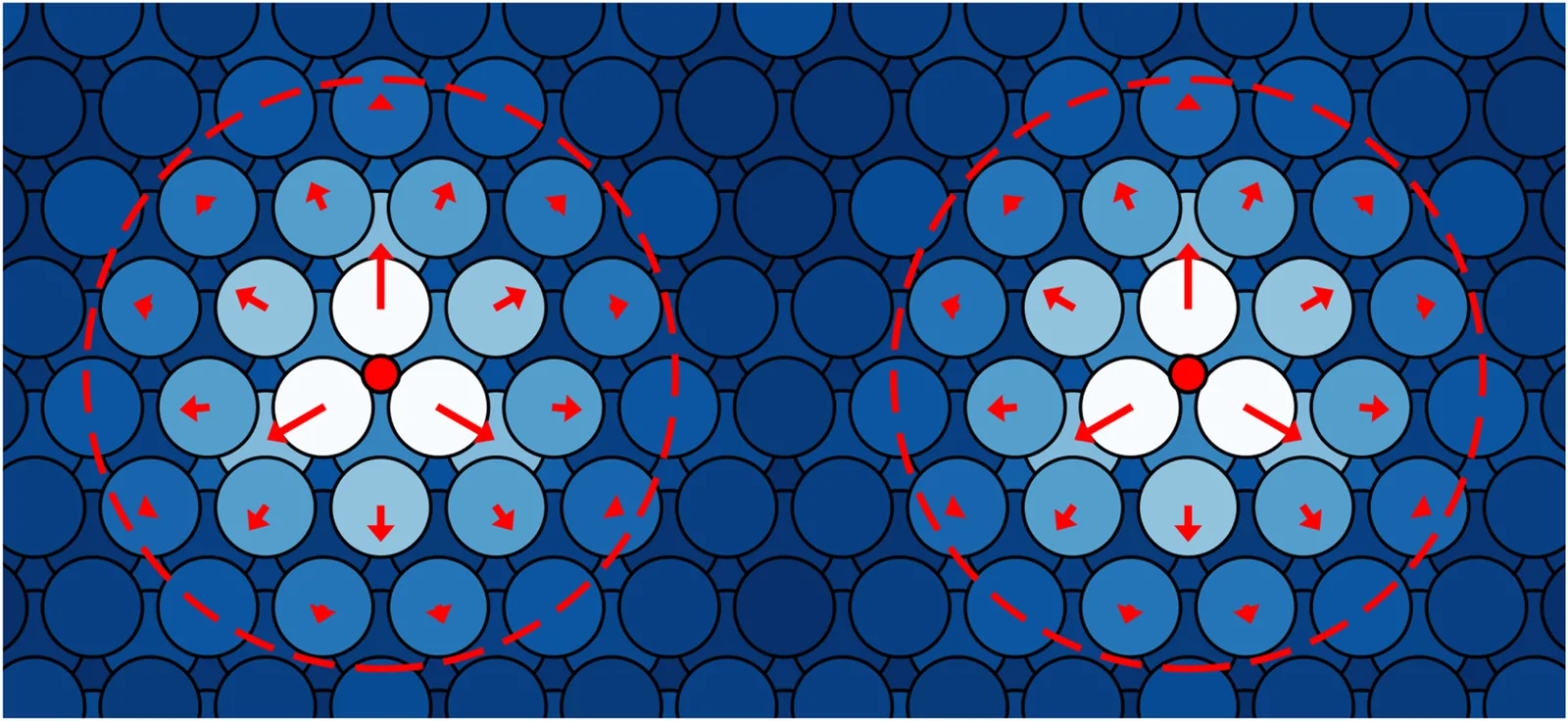

## 1 Introduction

Many reactions in heterogeneous catalysis proceed under conditions with high adsorbate coverages, where interactions between the adsorbates are important. These lateral interactions affect the kinetics of elementary processes and may drive the formation of ordered adsorbate structures during the reaction, which may dramatically enhance or suppress catalytic activity.^1–3^ Developing a quantitative understanding of lateral interactions is therefore essential for establishing predictive theories for catalytic reactions.

Lateral interactions can originate from electronic, electrostatic or elastic interactions^4^ acting either directly through space or indirectly through mediation by the surface.^4^ Both repulsive and attractive interactions have been observed in experiments that measure the adsorption energy of a molecule as a function of (co-)adsorbate coverage on the surface.^5–8^

Oxygen atoms on Pt(111) are a particularly interesting system for the study of lateral interactions. From low energy electron diffraction (LEED) and scanning tunneling microscopy (STM) experiments we know that ordered islands of a (2 × 2) overlayer structure form below saturation coverage,^9–11^ which has been taken as evidence of attractive interactions at third-nearest neighbor (3NN) distances and repulsive interactions at smaller distances.^10^ While theory and experiment agree on the existence of repulsive interactions between oxygen atoms at small distances,^9,12,13^ there is still controversy about the interaction energy at 3NN sites. Previous theoretical reports concluded repulsive^12^ and attractive interactions^13,14^ with various interaction strengths, depending strongly on the computational details and the method used for analysis. These studies also revealed the influence of long-range adsorbate induced strain.^12,13^

Of course, O-atom adsorption distorts the Pt surface structure in its vicinity. More interestingly, this distortion radiates outward and may influence distantly adsorbed O-atoms. DFT-based models capable of describing this effect have been developed and required interaction energy terms up to the 10NN.^13^ But computational cost restricted DFT calculations to using a (4 × 4) unit cell.^13–15^ The obvious question arises: can DFT data derived from calculations with a small simulation cell accurately describe adsorbate–adsorbate interactions influenced by long range strain?

In this work, we present DFT results using unit cells as large as (8 × 8) that accurately describe the lateral interactions between oxygen atoms adsorbed to face-centered cubic (fcc) sites of Pt(111). We show that oxygen adsorption changes the Pt-lattice structure at remarkably large distances and that this long-range adsorbate-induced surface strain is crucial to an accurate description of lateral interactions in this system. Surface strain influences oxygen interaction energies in two ways. First, attractive interactions at 3NN sites only arise for calculations based on large (8 × 8) unit cells, where interaction with periodic images becomes unimportant. Second, adsorbate induced surface strain causes repulsive interactions between oxygen atoms up to distances as large as 14 Å.

We also explored how the long-range repulsive interactions present in this system influence the morphology of oxygen islands. From kinetic Monte Carlo (kMC) simulations we find that without long-range interactions, islands are compact and circular. Including long range repulsion elongates and shrinks the islands, such that they more closely resemble images of islands obtained with STM.

## 2 Methods

### 2.1 DFT calculations

The electronic structure calculations shown in this work were performed using Vienna *Ab initio* Simulation Package VASP 6.4.3.^16–18^ A plane-wave basis cutoff of 500 eV and the Methfessel–Paxton method of second order^19^ with a smearing width of *σ* = 0.15 eV were employed. We studied the effect of the exchange correlation functional on the results for the PBE,^20^ RPBE,^21^ PBE-TS^22^ and RPBE-D3^23^ functionals.

We used several supercell representations of the Pt(111) surface ranging from (2 × 2) to (8 × 8). To account for the changing unit-cell size, we employed a (*N*_*k*p_ × *N*_*k*p_ × 1) *k*-point grid of the Brillouin zone, centered by the Monkhorst and Pack method,^24^ where *N*_*k*p_ was chosen such that the product of *N*_*k*p_ and the length of the unit cell in the *x* direction was always greater than 80 Å. This procedure ensured *k*-point convergence across the different unit-cell sizes.

Each slab had 15 Å of vacuum space along the surface normal direction and four layers. The bottom layer was fixed to bulk coordinates based on optimized lattice constants (PBE: 3.967 Å, RPBE: 3.990 Å, PBE-TS: 3.932 Å, RPBE-D3: 3.938 Å). All other layers were optimized using the conjugate gradient algorithm until the norms of their forces fall below 0.02 eV Å^−1^. The electronic structure was optimized until the total free energy change between two steps was smaller than 10^−5^ eV.

### 2.2 Kinetic Monte Carlo simulations

The kMC simulations of O* on Pt(111) were performed with Zacros 4.0.^25^ The Pt(111) surface was modeled by a (40 × 40) lattice of hexagonal symmetry with periodic boundary conditions and a nearest-neighbor Pt distance *a*_0_ = 2.82 Å. The movement of the O* atoms was limited to fcc sites on the surface, justified by the 0.5 eV fcc site preference over hcp sites.^26^ We assumed an Arrhenius temperature dependence of the hopping rate constant with an activation energy of 0.43 eV and a prefactor of 5 × 10^−9^ s^−1^, consistent with previous STM work.^10,27^ The interaction energy of adsorbates was computed using a graph theory-based cluster expansion approach. The interaction energy changes the hopping rate based on a Brønsted–Evans–Polanyi approach as implemented in Zacros^28,29^ using a proximity factor of 0.5. Note that we study equilibrium properties in this work and the value of the hopping rate constant does not influence the presented results.

Kinetic Monte Carlo simulations of atomic oxygen adsorbed on the Pt(111) surface were performed for a surface coverage of O*/Pt = 0.11 ML for surface temperatures of 100 K and 200 K. For each condition 10 trajectories of 2 × 10^5^ hopping events were calculated starting from the random distribution of adsorbates on the lattice. Observing the average energy as a function of time we found that the equilibrium state was reached after 10^5^ events even for the lowest simulated temperatures. From the equilibrium configurations 10^3^ geometries were used for statistical analysis.

To quantify the structural properties of adsorbed oxygen atoms on the lattice we used the radial distribution function (RDF), cluster size distribution, and a new quantity we introduced here, the adsorbate accessibility. The RDF was calculated by counting the number of occupied sites in the neighboring shells for an adsorbate and normalizing it by the corresponding value for a 2D lattice ideal gas. The cluster size distribution was calculated by means of *k*-dimensional tree (*K*-d tree) method combined with UnionFind package as implemented in the SciPy.^30^ To investigate the effect of a cluster expansion Hamiltonian on the structural properties of islands, we introduced the accessibility *α*_*i*,*C*_ of adsorbate *i* for a configuration (lattice snapshot) *C* defined as the number of paths that exist to access the adsorbate (*i.e.*, to become its nearest neighbor). Specifically, a path was considered to be ‘free’ if the corresponding first and third neighboring sites were unoccupied. Due to the hexagonal symmetry of the fcc(111) lattice, the accessibility ranges from 6 for an isolated adsorbate to 0 for an interior adsorbate of an island. For each configuration *C* belonging to the pool of simulated equilibrium adsorbate geometries we calculated a histogram of accessibilities1
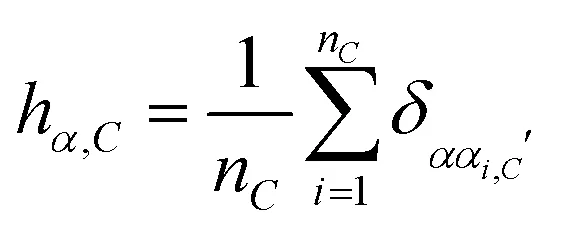
giving the fraction of adsorbates (out of *n*_*C*_) possessing accessibility *α* for configuration *C*. Here, *δ*_*αα*′_ is a Kronecker delta. The mean value of eqn (1)2
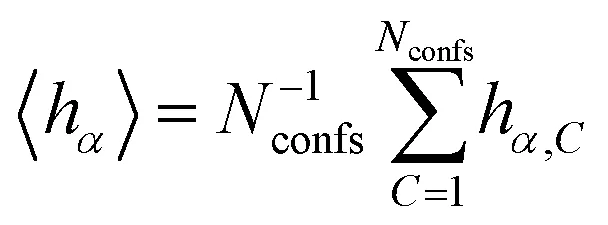
over total number of equilibrium configurations *N*_confs_ and its standard deviation were used to produce the plots in Fig. 6.

## 3 Results

### 3.1 Long-range interactions between adsorbed oxygen atoms on Pt(111)

Following previous work,^4^ we define the total interaction energy *E*_int_ of a structure containing *n* oxygen atoms by eqn (3)

3 *E*_int_ = *E*(slab + *n* × O*) − *nE*(slab + O*) + (*n* − 1)*E*(slab)

where *E*(slab + *n* × O*) is the energy of a structure with *n* oxygen atoms adsorbed simultaneously on the Pt simulation cell (slab), *E*(slab + O*) is the energy of a system with only a single adsorbed oxygen atom and *E*(slab) is the energy of the clean slab. Note that to correctly remove the energy of spectator Pt atoms, the third term adds back the (*n* − 1) slab energies subtracted in the first two terms.

For this definition of the interaction energy to be valid, *E*(slab + O*) must not contain any interaction between oxygen atoms arising from periodic images used in the DFT total energy calculations. Note that *E*_int_ > 0 for repulsive interactions and *E*_int_ < 0 for attractive interactions.

We calculated *E*_int_ for oxygen atoms in 1NN, 2NN and 3NN sites for (4 × 4) and (5 × 5) slab sizes with four different GGA functionals. The results in Table 1 imply repulsive interactions at all distances, exhibiting only a very weak dependence on the choice of functional.

**Table 1:** DFT-derived interaction energies between oxygen atoms at first-, second- and third-nearest neighboring (NN) sites. Results are shown for four different GGA functionals and for two unit-cell sizes. Positive values indicate repulsive interactions

Functional	*E*_int_ (meV)		
	1NN	2NN	3NN
**(4 × 4) unit cell**			
RPBE	195	96	9
PBE	197	97	14
RPBE-D3	201	96	27
PBE-TS	198	97	18

**(5 × 5) unit cell**			
RPBE	163	61	28
PBE	164	60	30
RPBE-D3	159	52	29
PBE-TS	162	54	25

**Fig. 1:**
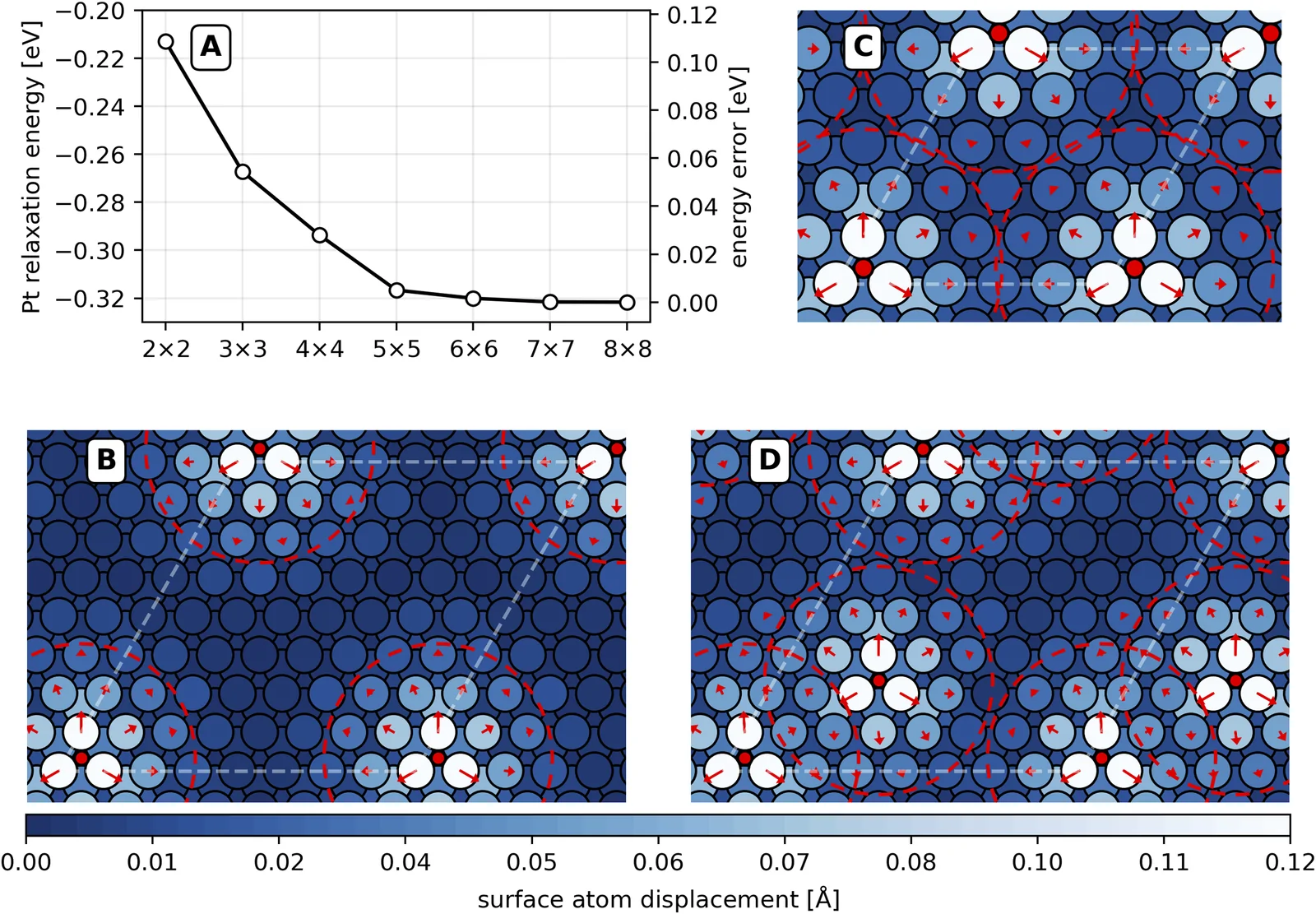
Adsorbate induced surface strain causes long-range lateral interactions between oxygen atoms on Pt(111). (A) Surface relaxation energy when adsorbing a single oxygen atom to fcc sites of Pt(111) for various unit-cell sizes. The right *y*-axis shows the energy difference with respect to the (8 × 8) calculation. (B) Optimized structure of oxygen adsorption on a (8 × 8) cell. O* atoms are colored red, Pt atoms are colored from dark blue to white, according to their displacement upon O atom adsorption. The surface displacement magnitude is shown in the color bar at the bottom of the figure. The red arrows indicate the direction of Pt atom movement in the plane of the metal surface. Dashed red circles of 14.4 Å diameter are drawn around each oxygen atom to indicate the sphere of influence due to surface strain. (C) and (D) are the same as (B), but for a (5 × 5) unit cell and for two oxygen atoms in 6th nearest-neighboring sites on an (8 × 8) unit cell, respectively.

To investigate the effect of long-range strain on lateral interactions, we performed additional DFT calculations using the RPBE functional on unit-cell sizes ranging from (2 × 2) to (8 × 8). In the calculations of Fig. 1(A), we first optimized the O* atom’s geometry on a frozen Pt lattice and investigated the effect of subsequently relaxing the upper 3 layers of Pt atoms. The magnitude of this relaxation energy increases with cell size and converges. These calculations show explicitly that the energy of an isolated O* atom—*E*(slab + O*) from eqn (3)—is not accurately described by the smaller unit-cell sizes. The use of small unit-cell sizes leads to an energetic error associated with *E*(slab + O*), which is shown on the right *y*-axis of Fig. 1(A).

Adsorbate induced strain radiating outward to neighboring periodic images is the cause of the cell-size dependence of the surface relaxation energy. Fig. 1(B) shows the influence of long-range strain pictorially—note the length of the red arrows, which indicates the magnitude of Pt atom displacement upon adsorption. The chemisorption of the O atom induces an expansion of the Pt(111) surface structure that decays only slowly with distance—at 7.2 Å distance, platinum atoms move by more than 0.020 Å. We take this value as the characteristic radius of strain around each oxygen atom; see the red dashed circles in Fig. 1(B).

For smaller unit cells whose size is similar to or smaller than twice the radius of strain, oxygen atoms experience interactions with their periodic images through surface strain—Fig. 1(C) shows results with a (5 × 5) unit cell. Here, all Pt surface atoms are displaced by more than 0.010 Å from their adsorbate-free positions, resulting in an energy error. The deviation is even more pronounced for smaller unit-cell sizes and explains why the interaction energies at the 3NN distance listed in Table 1 are all repulsive.

Using large unit cells, we see that the adsorbate induced strain field is responsible for long-ranged repulsive interactions between oxygen atoms. Fig. 1(D) shows the interaction of an oxygen atom with its 6NN computed accurately on an (8 × 8) cell. Here, oxygen atoms experience a repulsion of +13 meV, an interaction that is mediated by displacements of the Pt atoms between them.

### 3.2 Many body expansion for the interaction between adsorbed oxygen atoms

To carry out kinetic Monte Carlo simulations of O*-atom island formation, it is convenient to first expand the interaction energy in many-body terms, known as a cluster expansion.4
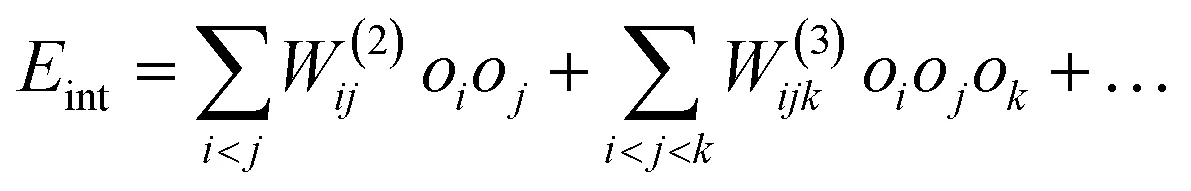
Here *W*^(2)^ and *W*^(3)^ describe the magnitude of two- and three-body interactions, and *o*_*i*_ is 1 if site *i* is occupied and zero otherwise. We truncate the expansion after the three-body terms, consistent with previous work on this system that showed negligible four-body energy terms.^14^ We will show below that the three-body terms are also negligible.

**Fig. 2:**
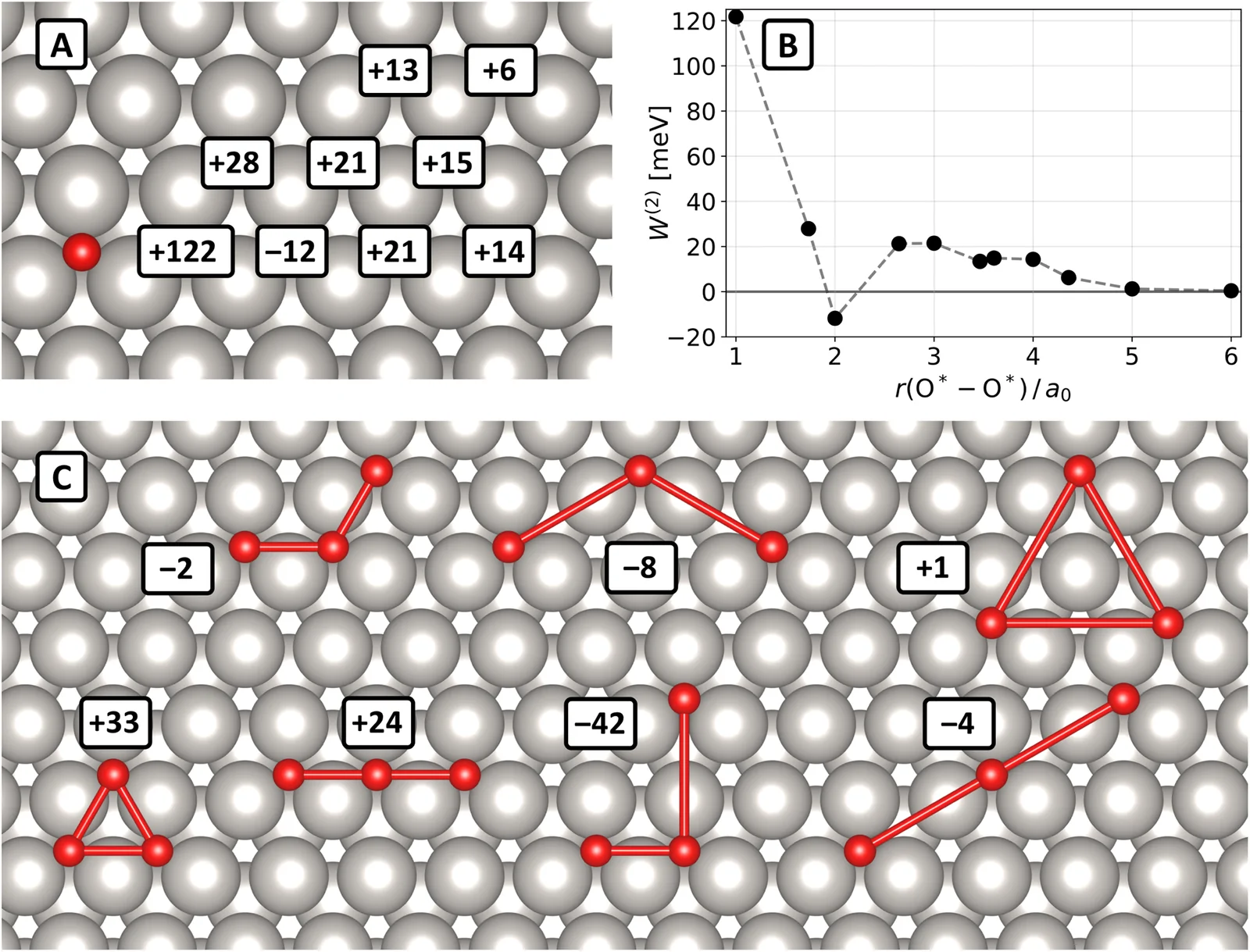
Interaction energy coefficients between oxygen atoms (O*, red circles) adsorbed to face-centered cubic sites of Pt(111). (A) Two-body interaction energy coefficients *W*^(2)^ (in meV) are indicated in the text boxes at the respective positions of the second oxygen atom. (B) Two-body interaction energy coefficients *W*^(2)^ as a function of O*–O* distance *r* in units of the Pt–Pt distance on the surface *a*_0_. Two data points at *r*/*a*_0_ = 5, 6 were obtained from calculations of a single oxygen atom on a (5 × 5) and (6 × 6) unit cell, other points represent values shown in panel (A). The dashed grey line is drawn to guide the eye. (C) Configurations used for calculations of three-body interaction energy coefficients *W*^(3)^. Results are given in meV in the text boxes.

In light of the slow convergence with unit-cell size shown above, we used an (8 × 8) unit cell to compute interaction energies up to the 9NN and to fully map the strain field induced by adsorbed oxygen atoms. This limits the number of interactions across periodic boundaries, ensuring that eqn (4) can be used to accurately determine each coefficient (*W*^(2)^_*ij*_ or *W*^(3)^_*ijk*_) from a single DFT calculation. Specifically, we calculated energies of 9 structures with two oxygen atoms and 7 structures with three oxygen atoms in fcc sites of the Pt(111) (8 × 8) unit cell. Fig. 2(A) shows the results of the two-body calculations, where the reference oxygen atom is shown as a red circle and text boxes indicate the value of *W*^(2)^ (in meV) at various neighbor positions. All two-body interactions with the exception of the 3NN are repulsive. This is shown more clearly in Fig. 2(B), where *W*^(2)^ is plotted against the inter-O*-atom distance *r*.

Fig. 2(C) shows structures with three oxygen atoms from which we calculated three-body coefficients. The values of the coefficients *W*^(3)^ are given in the text boxes close to the structures.

### 3.3 Effects of long-range repulsive interactions on oxygen island formation

**Fig. 3:**
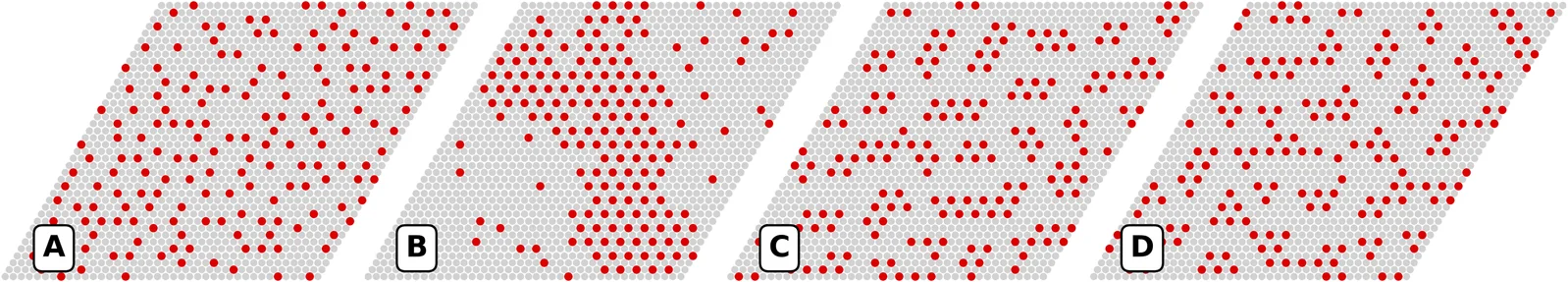
Effect of lateral interaction on equilibrium kMC configurations of 0.11 ML O* atoms adsorbed (red dots) at fcc sites on Pt(111) at 100 K. Snapshots of configurations on a 40 × 40 lattice with periodic boundary conditions are shown for inclusion of various types of lateral interactions: (A) two-body interactions up to 2nd nearest neighbor; (B) interactions of panel (A) plus attractive interactions at 3rd nearest neighbor distance; (C) interactions of panel (B) plus long range repulsive two-body interactions up to 9th nearest neighbor; (D) all two- and three-body interactions discussed in the text.

To investigate how long-range strain affects oxygen island formation, we performed kMC simulations (as described above in Section 2.2) at a surface temperature of 100 K and a coverage of O*/Pt = 0.11 ML. Fig. 3 shows representative configurations taken from kMC simulations, and how they change when increasing the number of terms in many-body expansion. In panel (A) only 1NN and 2NN lateral interactions are included and since both are repulsive, their effect can be seen only by the absence of pairs of atoms at these distances. When including attractive 3NN interactions, O* atoms form ordered islands—Panel (B). These islands are compact and approximately round to the extent permitted by the hexagonal lattice. Panel (C) shows the effect of including 1NN to 9NN two-body interactions—these interactions dramatically alter both the sizes and the shapes of the islands. Smaller linear clusters are now present and the large “circular” clusters are absent. In fact, these images bear an uncanny similarity to STM images of O atoms adsorbed on Pt(111).^10,11^ Finally, panel (D) shows the results of calculations that added three-body interaction terms.

**Fig. 4:**
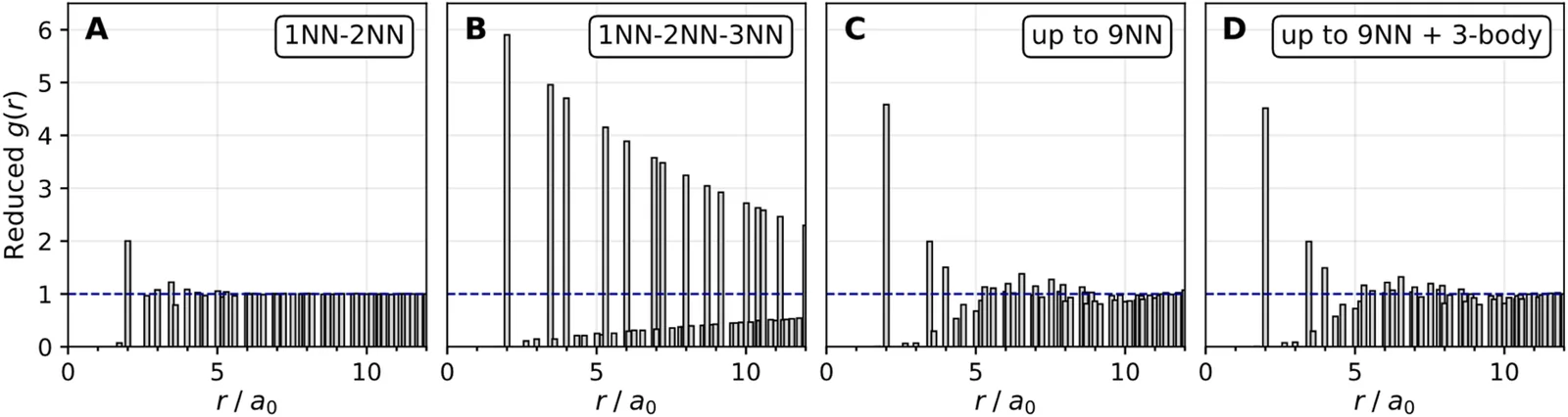
Radial distribution functions (RDF) averaged over 10^3^ equilibrium kMC configurations for the same conditions as Fig. 3. The dashed horizontal line indicates the value of the RDF expected for an ideal lattice gas. Note the dramatic change in going from two-body interactions up to 3rd nearest neighbor (3NN) in (B) to inclusion of long range repulsive two-body interactions in (C) and the minor additional effect of also including three-body interactions in (D).

While the images of Fig. 3 are representative, a quantitative characterization of the islands requires statistics on many configurations, for example to obtain radial distribution functions (RDFs) and cluster size distributions. Fig. 4 shows RDFs averaged over 10^3^ kMC configurations for the 4 interaction-cases of Fig. 3. Panel (A) shows results when only 1NN and 2NN interactions were included—this assumption completely eliminates the 1NN peak and reduces the 2NN peak to near zero. Panel (B) shows results after adding the 3NN attractive interaction—the RDF is completely different. The 3NN peak increased by more than a factor of 2 and many additional long-range peaks also increase reflecting the formation of large compact islands. Including the long-range repulsive interactions—panel (C)—is equally striking. A large peak at the 3NN distance is seen indicating ordered (2 × 2) islands, but the long-range order nearly disappears. Including three-body interactions—panel (D)—does not significantly change the RDF compared to panel (C). Note that we have normalized the RDFs to one for an ideal 2D-lattice gas. In the non-interacting case, not shown, the RDFs were equal to one at all distances, confirming that the adsorbates behaved as an ideal lattice gas.

**Fig. 5:**
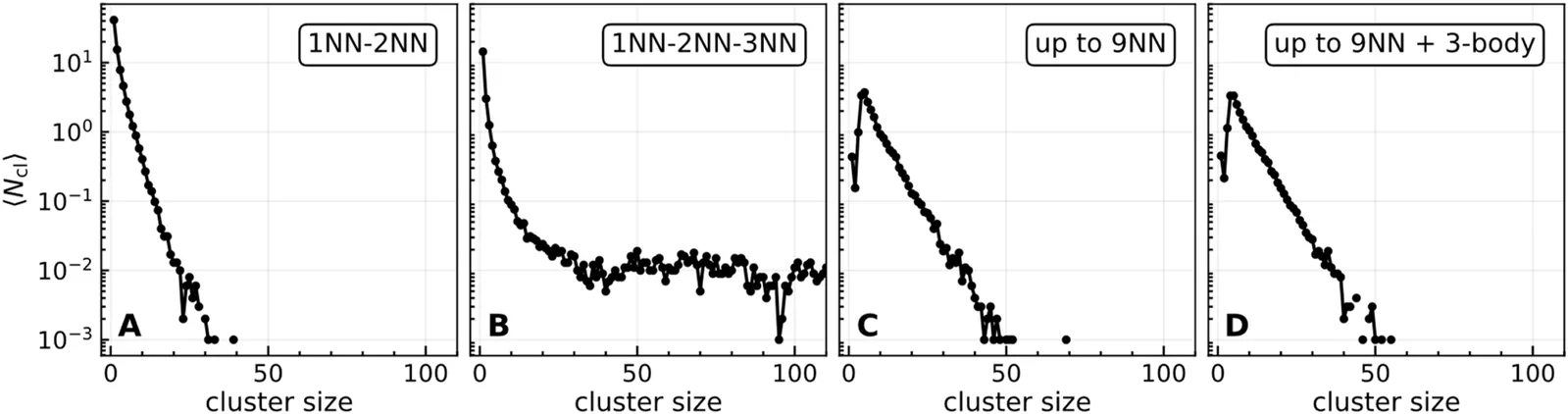
Cluster size distributions averaged over 10^3^ equilibrium kMC configurations for the same conditions as Fig. 3. Note the dramatic change in going from two-body interactions up to 3rd nearest neighbor in (B) to inclusion of long range repulsive two-body interactions in (C) and the minor additional effect of also including three-body interactions in (D).

Fig. 5 displays cluster size distributions for the 4 interaction cases shown above. For the 1NN–2NN repulsive-only interaction case in panel (A), the cluster size distribution is largest for a cluster size of one (isolated atom) and decays roughly exponentially as cluster size increases. Inclusion of attractive interactions at 3NN distance in panel (B) resulted in a very broad plateau in the cluster size distribution due to the formation of large O*-atom islands. Including the long-range repulsive interactions in panel (C) changes the distribution again in a striking way. Here, there is a peak in the cluster size distribution for clusters of 4 or 5 atoms followed by an exponential decay. The very broad plateau of larger clusters is completely absent. The cluster size distributions do not change when including three-body interactions, see panel (D).

The radial distribution functions and cluster size distributions are good metrics for the degree of long-range order and island size; however, they do not address reactive accessibility—O* atoms located deep within islands may not be accessible to reactive co-adsorbates—or site-specific reactivity—atoms on island perimeters may be more reactive than those at the interior (or *vice versa*). It is therefore useful to define a steric metric that describes the shapes of islands.

We define the accessibility of an O* atom as the number of paths a co-adsorbate has to potentially react with it. An isolated atom has an accessibility of 6—there are 6 directions of attack on the hexagonal fcc(111) lattice. These 6 directions are determined by the 1NN and 3NN sites around the adsorbate. We consider each direction to be blocked if either 1NN or 3NN, or both 1NN and 3NN sites are occupied. The resulting accessibility equals 6 − *N*_bl_, where *N*_bl_ is the number of blocked directions. O* at the interior of a (2 × 2) island will have an accessibility of 0, see also Section 2.2.

**Fig. 6:**
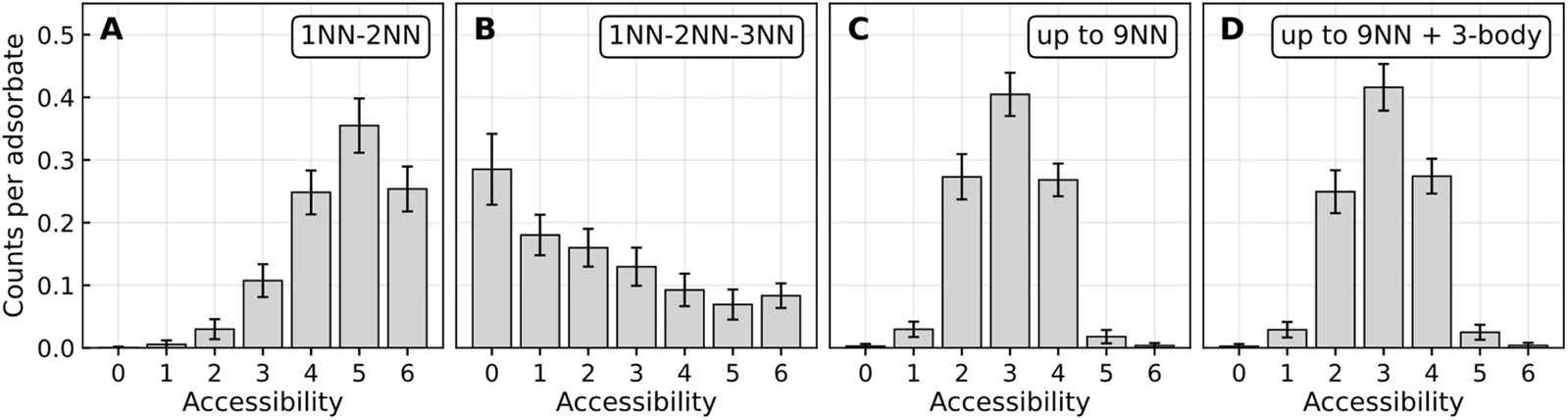
Accessibility histogram of adsorbate configurations averaged over 10^3^ equilibrium kMC configurations for the same conditions as Fig. 3. The error bars indicate 1*σ* standard deviations of the thermal fluctuations.

A histogram of accessibilities averaged over 10^3^ equilibrium kMC configurations for the different interaction cases under consideration is shown in Fig. 6. If only 1NN and 2NN interactions are considered, the accessibility is high with 6, 5, and 4 being the most likely accessibility. Adding the attractive 3NN interaction results in compact islands and the most probable accessibility is 0. If we include long-range repulsive 2-body interactions up to 9NN, the accessibility changes dramatically. Now the most probable accessibility is 3. Interestingly, long-range interactions decrease the probability of an adsorbate having an accessibility of 6 – *i.e.* they decrease the probability of finding isolated O*. This is explained by the fact that at the coverage used here (0.11 ML), there are few possibilities to create isolated atoms that avoid the repulsive long-range 4NN to 9NN interactions, and it becomes energetically more favorable for the adsorbate to bind to an existing island. Including 3-body interactions (panel D), does not have a significant effect on the accessibility, consistent with the similarity of the snapshots shown in Fig. 3C and D.

## 4 Discussion

We have presented a fully *ab initio* treatment of the strain field induced around an adsorbed oxygen atom at a Pt(111) surface and investigated how it influences adsorbate–adsorbate interactions. Based on DFT-GGA calculations we found that larger unit cells than previously used^12–15^ were needed to converge the results. The need to use these large unit cells reflects the long range over which an adsorbate may induce lattice distortions. To describe the characteristic size of this effect, we defined a radius of strain which, for oxygen atoms on Pt(111), is about 7 Å. This means that adsorbed oxygen atoms separated by 14 Å experience a repulsive interaction meditated by the distorted lattice of Pt atoms between them. We have also derived two-body interaction energy coefficients up to the 9NN distance as well as three-body interactions. The use of a DFT simulation cell of (8 × 8) enabled us to define an interaction free reference energy *E*(slab + O*) of a single oxygen atom adsorbed to Pt(111), see eqn (3), and allowed us to determine the two- and three-body energy coefficients directly from the DFT calculations. This ensures that adsorbate–adsorbate interactions are described well at short and long range.

Previous work based on small-cell DFT computations arrived at similar results to those shown in Fig. 2(B), but determined interaction energy coefficients *W*^(2)^_*ij*_ and *W*^(3)^_*ijk*_ by an optimization procedure using many DFT energies generated with a (4 × 4) unit cell.^13,15^ Here, long-range interactions between oxygen atoms residing in different unit cells, that is across periodic boundaries, are included. This procedure is, however, not without difficulties. The choice of how many different structures and which energy terms to add to the cluster expansion can significantly influence the values of coefficients in the interaction energy expansion,^13,15,28,31^ displaying cross-correlation between them. Furthermore, the method uses DFT data not converged with respect to unit-cell size and the number of independent configurations that can be created using a small unit cell limits the number of terms in the multi-body expansion that can be probed. Despite these potential problems, the results of that work are similar to those presented here; however, the fitted many-body expansion places greater importance on three-body interactions. Our results using an (8 × 8) cell show that the three-body terms are unimportant, raising the question: could the cluster expansion approach using a (4 × 4) unit-cell produce attractive interactions if only two body terms were used?

**Fig. 7:**
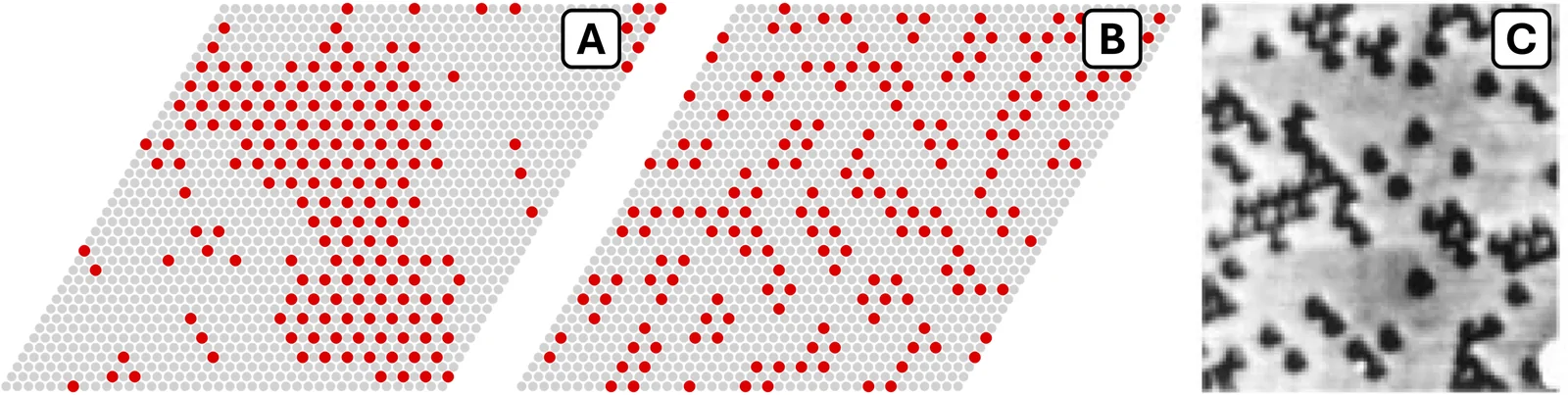
Comparison of oxygen island shapes on Pt(111). (A) and (B) are snapshots of kMC simulations at 100 K at 0.11 ML. Panel (A) includes interactions up to the third-nearest neighbor, (B) includes all two- and three-body interactions. Panel (C) is an STM image at 205 K; figure panel reprinted with permission from J. Wintterlin, R. Schuster and G. Ertl, *Phys. Rev. Lett.*, 1996, **77**, 123. Copyright 1996 by the American Physical Society.

To understand the implications of long-range interaction on the formation of oxygen islands, we used the many-body expansion established in this work to perform kMC simulations of equilibrium oxygen island structures and performed a statistical analysis of their sizes and shapes. This analysis showed that the long-range repulsive strain field exerts a profound influence on the morphology and stability of adsorbate islands and that long-range strain is more important than three-body interactions. The most intuitively obvious shape of an oxygen island—round and compact as shown in Fig. 7(A)—is rendered impossible by the strain induced repulsion. In fact, only a residual short-range attraction in 3NN sites makes islands possible at all. The interplay between these short-range attractions and long-range repulsions leads to small and elongated islands—Fig. 7(B)—that resemble those seen in STM measurements,^10,11^ see for example Fig. 7(C). This is easily understood. Since 4NN to 9NN two-body interaction energy coefficients are repulsive, the adsorbed layer restructures to minimize the number of atoms at 4NN to 9NN positions. By contrast, if these repulsive interactions are not included in the kMC simulations, the oxygen islands appear round and compact, inconsistent with STM images.

The interaction energy between oxygen atoms at 3NN sites is a crucial parameter determining the formation of (2 × 2) islands, whose existence is known from experiments.^9–11,27^ However, theoretical values strongly depend on the details of the DFT calculation, unit-cell size and method used to extract the interaction energy terms. Reported results include −16 meV,^13,28^ −6 meV^14^ and +9 meV,^12^ while the experiment results suggest a value of −25 meV.^9,27^ Previous work concluded that surface strain causes intrinsic attractive interaction (−23 meV) between oxygen atoms adsorbed in 3NN sites to become repulsive (+9 meV).^12^ We showed here that this is an artifact resulting from the definition of the interaction energy, which in ref. 12 was taken as the energy difference between oxygen atoms separated by the 3NN distance and single oxygen atoms on a (4 × 4) unit cell.^12^ This definition only works if the oxygen atoms are free of interactions with their periodic images. However, when surface atoms are allowed to relax in such a small unit cell, surface strain causes interactions between oxygen atoms and their periodic images. See Table 1 and Fig. 2(A). When using a sufficiently large unit cell—an (8 × 8) cell—these artificial interactions are avoided and DFT yields attractive interactions (−12 meV) between adsorbed oxygen atoms at 3NN sites.

The attractive interaction found here is slightly too weak to reproduce island size distributions at the temperature of the STM experiment in Fig. 7 (205 K). We accounted for this difference by scaling the temperature of our simulations presented in Fig. 7 by the ratio of the experimental attraction and the attraction found here (25/12 ≈ 2). To further explore this, we also performed simulations at 200 K with −25 meV attraction in 3NN sites while keeping all other two- and three-body interactions constant. These simulations reproduce the STM images in a similar way.

## 5 Perspective and future work

Adsorbed oxygen atoms on platinum surfaces play an important role in various heterogeneously catalyzed reactions, including the Ostwald process, reactions in the automotive catalytic converter, and the electrochemical oxygen reduction reaction. This work identifies challenges and raises important questions for computational modeling of such reactions. Modeling based on the use of (2 × 2) or (3 × 3) unit cells, as is common, may be strongly influenced by adsorbate-induced strain fields. We can use the results of this work to get some idea of the magnitude of the errors introduced by this practice. For example, if we consider a (3 × 3) unit-cell for the O*–Pt system, we see that the strain-field induced error is ∼50 meV. This is much larger than the uncertainty in the interaction energy associated with the choice of functional in underlying DFT calculations. This is illustrated in Table 1, where the standard deviation of the interaction energies obtained with four functionals is ∼5 meV. Clearly, in order to obtain the highest accuracy within DFT, reducing errors associated with the strain field is essential.

The implications of this work extend beyond the O*–Pt system, as other adsorbates may also induce surface strain upon adsorption. This is particularly likely for atoms and molecules that interact strongly with the surface, like C, N, OH or CO, all of which play a crucial role as intermediates in catalytic reactions. Now that calculations on large unit cells have become possible, it would be straightforward to find the radius of strain of many other adsorbates and explore its effect on lateral interactions. Studying other adsorbates would also help to investigate the physical origin of the phenomenon and its relation to the properties of the adsorbate.

In this work we have focused on lateral interactions on the Pt(111) surface. It would be very valuable to explore adsorbate-induced strain on more complex platinum surfaces, such as those with steps and defects, and on other metals to determine whether this effect is unique to platinum or a general phenomenon. It is also interesting to ask how adsorbate induced strain might affect rates of surface reactions. The adsorbate induced strain field may affect the adsorption energy of reactants and the energy of transition states differently, and therefore change the barrier heights of surface reactions. Additionally, the formation of ordered adsorbate structures, such as islands, creates varying local environments, which can significantly influence reactivity at elevated surface coverages. For instance, in many surface reactions, only the atoms at the island perimeters are accessible to other reactants. If the islands are circular, the number of atoms in the perimeter is proportional to the square root of the number of total atoms.^32^ Square root scaling laws have been used as approximate models for reactivity for systems that are subject to island formation.^33^ The change in island morphology due to long-range repulsive interaction observed in this work clearly would change these scaling laws.^34^ For islands with irregular-shaped boundaries, the accessibility parameter we have defined may be useful in estimating reactivity, but much further work is required to develop a general relationship. Furthermore, the heights of reaction barriers may depend on the configuration of atoms in the island relative to the atoms participating in the catalytic reaction, a very interesting issue for future work.

## Conflicts of interest

There are no conflicts to declare.

## Data Availability

The results of density functional theory calculations required to reproduce the results of this work are available at: F. Nitz, The effect of adsorbate induced surface strain on oxygen island formation on platinum surfaces [Data set], *Zenodo*, 2025, https://doi.org/10.5281/zenodo.17885846.
